# Percutaneous ultrasound-guided core needle biopsy for the diagnosis of cardiac tumors: Optimizing the treatment strategy for patients with intermural and pericardial cardiac tumors

**DOI:** 10.3389/fonc.2022.931081

**Published:** 2022-08-05

**Authors:** Ying Xie, Zhi-liang Hong, Yan-chun Zhao, Sheng Chen, Yu-cheng Lin, Song-song Wu

**Affiliations:** ^1^ Department of Hematology, Fujian Provincial Hospital, Shengli Clinical Medical College of Fujian Medical University, Fuzhou, China; ^2^ Department of Ultrasonography, Fujian Provincial Hospital, Shengli Clinical Medical College of Fujian Medical University, Fuzhou, China; ^3^ Department of Ultrasonography, Affiliated Fuzhou First Hospital of Fujian Medical University, Shengli Clinical Medical College of Fujian Medical University, Fuzhou, China

**Keywords:** ultrasonography, cardiac tumors, pericardial tumors, percutaneous ultrasound guidance, puncture

## Abstract

**Objective:**

The aims of this study are to investigate the clinical value and practical safety of ultrasound-guided percutaneous core needle biopsy on diagnosing cardiac tumor and to discuss the treatment strategy for cardiac intermural and pericardial tumors.

**Methods:**

The clinical data were retrospectively collected for patients with intermural and pericardial cardiac tumors. The patients were divided into groups of surgical resection, surgical resection after obtaining pathological tissue by PUS-CNB, and/or radiotherapy according to the treatment modality. Ultrasound-guided aspiration biopsy was divided into cardiac tumor biopsy and extracardiac lesion biopsy according to patient conditions. The surgical time was recorded, and the safety and clinical application value of PUS-CNB for the diagnosis of cardiac tumors were evaluated in terms of complications and satisfaction with pathological sampling.

**Results:**

A total of 18 patient cases were collected, and PUS-CNB of cardiac tumors was performed in 8 cases, with sampling times averaging 15.6 ± 3.0 min. Four cases of cardiac tumors combined with extracardiac tumors were biopsied, with puncture times averaging 13.0 ± 2.9 min. All 12 biopsied patients had no postoperative complications. Except for 1 failed biopsy, the biopsies were successful and the pathological results were consistent with the clinical diagnosis with a satisfaction rate of 91.7%. Except for two cases of surgical resection, the rest were considered for conservative treatment. Surgical resection and/or biopsy were performed in six cases, and two cases were aggravated after surgery. The final pathology of all 17 cardiac tumors was malignant.

**Conclusion:**

PUS-CNB is safe and effective, providing a simple and undemanding method for accurate diagnosis of cardiac intermural and pericardial tumors while avoiding unnecessary open-heart surgery.

## Introduction

Cardiac tumors are uncommon in clinical practice, with an incidence of about 0.05%–0.20% (1). About 90% of cardiac tumors are benign (2, 3), among which cardiac myxoma account for 70%–80%, and 80%–90% of that occur in the left atrium (4–6). Malignant tumors of the heart are relatively uncommon. According to the location, cardiac tumors include those occurring in the heart and pericardium (7), and cardiac tumors are divided into intracavitary and intermural tumors according to the growth site (8). With the development of imaging technology, the combined applications of transthoracic or transesophageal echocardiography, magnetic resonance imaging (MRI), multidetector computed tomography (CT), and positron emission computed tomography (PET/CT) all have advantages in the differential diagnosis of cardiac tumors (9). Yet, imaging diagnosis is still not the gold standard of tumor histopathology, and tissue biopsy has important clinical value for accurate preoperative evaluation and personalized treatment.

The use of percutaneous ultrasound-guided core needle biopsy (PUS-CNB) for the diagnosis of cardiac tumors has rarely been reported in the past. In this paper, based on the previous study (10), the safety and clinical application value of ultrasound-guided percutaneous core needle biopsy in the diagnosis of intermural and pericardial cardiac tumors were further investigated by comparing and analyzing with surgical methods to provide a basis for clinical selection of appropriate treatment modalities and optimization of cardiac malignant tumor diagnosis and treatment strategies.

## Materials and methods

In this study, 8 male and 10 female patients, 24–87 years of age, with intermural and pericardial tumors were retrospectively collected from January 2012 to December 2021 at Fujian Provincial Hospital. Eight patients with intermural and pericardial tumors were biopsied with percutaneous ultrasound guidance, four patients with extracardiac lesions were biopsied with ultrasound guidance, and six patients’ cases involved surgical resection and/or biopsy. The surgical resection or biopsy was performed in the cardiac surgery department, and the ultrasound-guided percutaneous puncture biopsy was performed in the interventional ultrasound department by two interventionalists (S-SW and SC, who had 15 years of experience in interventional ultrasound). All patients in this study signed an informed consent form before the procedure.

The study included intermural and pericardial tumors, namely, tumors pertaining to the myocardial wall and partial extrusion convexly to the pericardium, and intra-pericardial neoplasms, including primary and secondary tumors. Inclusion criteria for ultrasound-guided biopsy of cardiac tumors were as follows (1): unknown clinical diagnosis with active cardiac mass determined by PET/CT, enhanced MRI and other imaging examinations and no known existence of extra-cardiac foci that were easier to biopsy; (2) a safe puncture route was available; and (3) patient condition that was able to tolerate percutaneous ultrasound puncture. Exclusion criteria were as follows: (1) cardiac mass without a safe puncture route, such as lesions located deeply in the myocardial wall or protruding into the cardiac cavity, there is a risk of penetrating the myocardial wall; (2) the heart lesions blocked by the sternum, ribs, or interfered by lung air, and still cannot be clearly displayed by using auxiliary methods; (3) hypervascular cardiac mass with large tortuous blood vessels on its surface; (4) preoperative imaging shows obvious necrosis inside the lesion, and it is estimated that the biopsy specimen is difficult to make a clear pathological diagnosis; (5) patients with severe coagulation dysfunction; and (6) comorbidity with severe breathing difficulties or restlessness, unable to cooperate with puncture.

A Philips iU22, GE Vivid 7 Dimension color Doppler ultrasonograph, C5-2 probe (2–5 MHz), and M4S probe (2–3.5 MHz) were used. An 18G fully automated biopsy gun (BARD Magnum, MN18-20, CR) and puncture stand were utilized.

### Puncture biopsy specifications and procedures

Patients were evaluated by the interventional team physicians and all had met the inclusion criteria before the procedure was performed. All patients were preoperatively checked with 12-lead ECG, transthoracic echocardiogram, chest radiograph, coagulation function, and routine blood tests.

The patient was placed in the left lateral or horizontal position, and the ultrasound probe was used to search for the heart lesion from the apical region, the intercostal space, and the parasternal area to observe the site, size, morphology, internal echo, and its relationship with the heart tissue.

After surface disinfection of the precordial area, spreading of the towel, and local anesthesia with 2% lidocaine, the probe was covered with a sterilized probe sleeve, and the puncture frame was placed. A suitable safe puncture point was found (usually the apex area as the main one), the 18G automatic biopsy gun was guided through the hole of the puncture frame, and the real-time ultrasound was observed during the needle tip passes through the skin and subcutaneous tissue to the lesion (note that the puncture path is in the direction of the needle entry parallel to the myocardial wall as much as possible). The puncture gun was then activated and the needle was withdrawn. To ensure the quality of the biopsy, each patient was punctured two or three times to obtain tissue strips of 15-20 mm in length, which were later fixed by 10% formaldehyde solution and sent to the pathology department. If a large amount of pericardial effusion was accompanied, catheter drainage was performed under ultrasound guidance at the same time. The needle tract was observed with ultrasound 5, 10, and 30 min postoperatively. If the patient had no discomfort and no pericardium or pleural effusion, the patient was sent back to the ward. If chest pain, palpitations, and pericardium and pleural effusion occurred during this period, electrocardiogram and transthoracic cardiogram were performed.

The treatment time (time from the beginning of ultrasound localization to the end of puncture for ultrasound-guided biopsy, and the operation time from the start of skin incision to the end of suturing) and complications were recorded, and the satisfaction of pathology sampling was used as an index to evaluate the success of biopsy. Satisfaction with pathology sampling was evaluated by whether there was sufficient amount of pathological tissue for the pathologist to obtain positive pathological results. The outcome of patients with different treatment modalities was followed up.

### Statistical analysis

Given the descriptive aim of the research, no formal statistical design has been established. Continuous variables were expressed as the mean ± standard deviation. Descriptive data are presented as a percentage of the entire number of patients.

## Results

### PUS-CNB of cardiac tumors

Eight cases of cardiac tumors were biopsied by ultrasound-guided percutaneous puncture, and except for two cases of diffuse intrapericardial occupancy, the maximum diameter of the remaining six cases ranged from 35 to 81 (54.7 ± 18.0) mm, among which 62.8% (5/8) of patients with cardiac tumors were accompanied by pericardial effusion and two cases received pericardial catheter drainage at the same time.

Puncture pathway and complication: the apex area of the fifth intercostal space was chosen as the puncture point in seven cases, and the parasternal part of the third intercostal space on the left side was chosen as the entry pathway in one case of base of cardiac tumor. The location of tumors is varied: three cases were intermural tumors and five cases were pericardial tumors. The puncture time ranged from 9 to 20 (15.6 ± 3.0) min. All patients tolerated the puncture with slight pain, and there was no serious bleeding (no significant change in the volume of pericardial effusion on re-examination) or infection after the operation.

**Figure 1:**
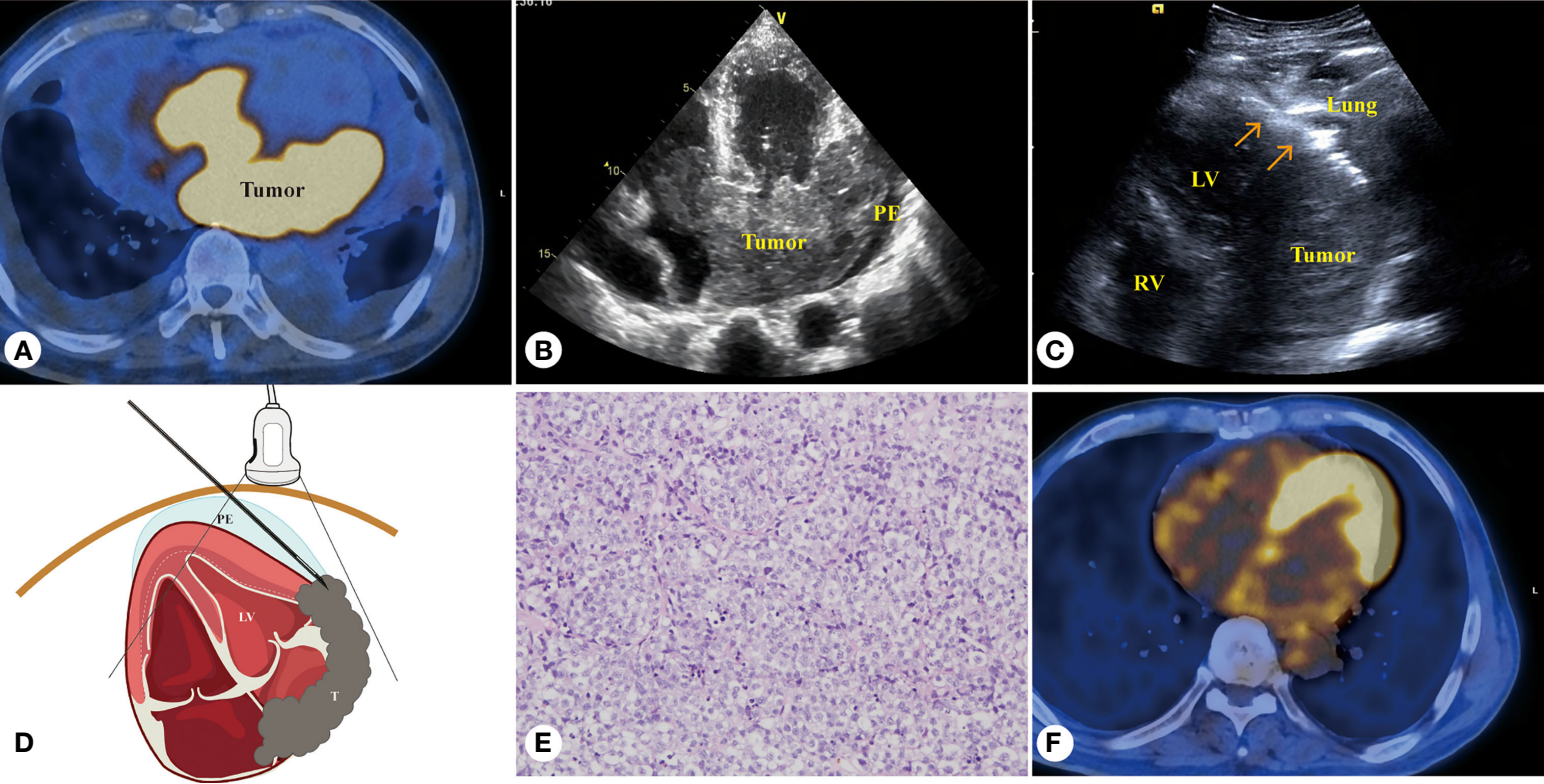
Sixty-five-year-old male patient, diffuse large B-cell lymphoma of the heart. **(A)** PET/CT revealed abnormal concentrated lesion (SUV 12.3–26.5) of the heart. **(B)** Echocardiography revealed the tumor invading the left atrial wall, atrial septum, atrioventricular valve, and right ventricle. **(C)** PUS-CNB of left atrial wall tumor (red arrow shows the biopsy needle is parallel with the myocardial wall). **(D)** Schematic diagram of PUS-CNB. **(E)** The microscopic features showed diffuse patches of medium to large atypical lymphocytes (H&E, × 200). **(F)** After 4 courses of chemotherapy, PET/CT showed that the lymphoma lesion disappeared. LA, left atrium; LV, left ventricle; RV, right ventricle; RA, right ventricle; T, tumor; PE, pericardial effusion.

**Figure 2:**
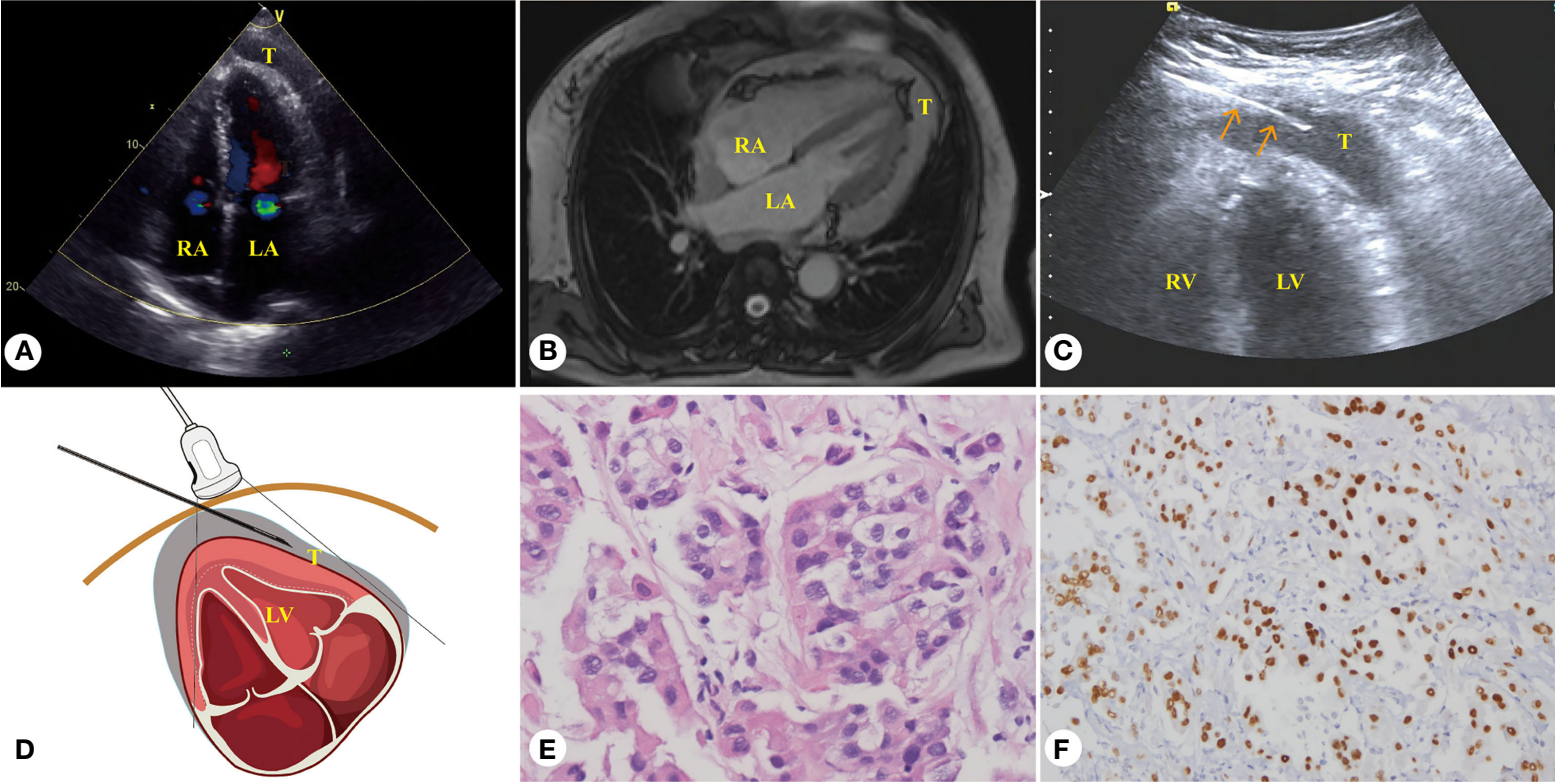
Sixty-year-old female patient, malignant mesothelioma of the pericardium. **(A)** Echocardiography revealed diffuse thickening lesion of the pericardium. **(B)** Axial conventional T2WI showed irregular diffuse long T2 signal lesions of the pericardium. **(C)** PUS-CNB of the apical pericardial hypoechoic lesions (red arrow shows that the biopsy needle is parallel with the myocardial wall). **(D)** Schematic diagram of PUS-CNB. **(E)** The microscopic features showed eosinophilic epithelioid cells arranged in a nested or glandular shape (H&E, × 400). **(F)** Immunohistochemically, the tumor cells nuclei were strongly positive for WT-1 (× 200). LA, left atrium; LV, left ventricle; RV, right ventricle; RA, right ventricle; T, tumor.

**Figure 3:**
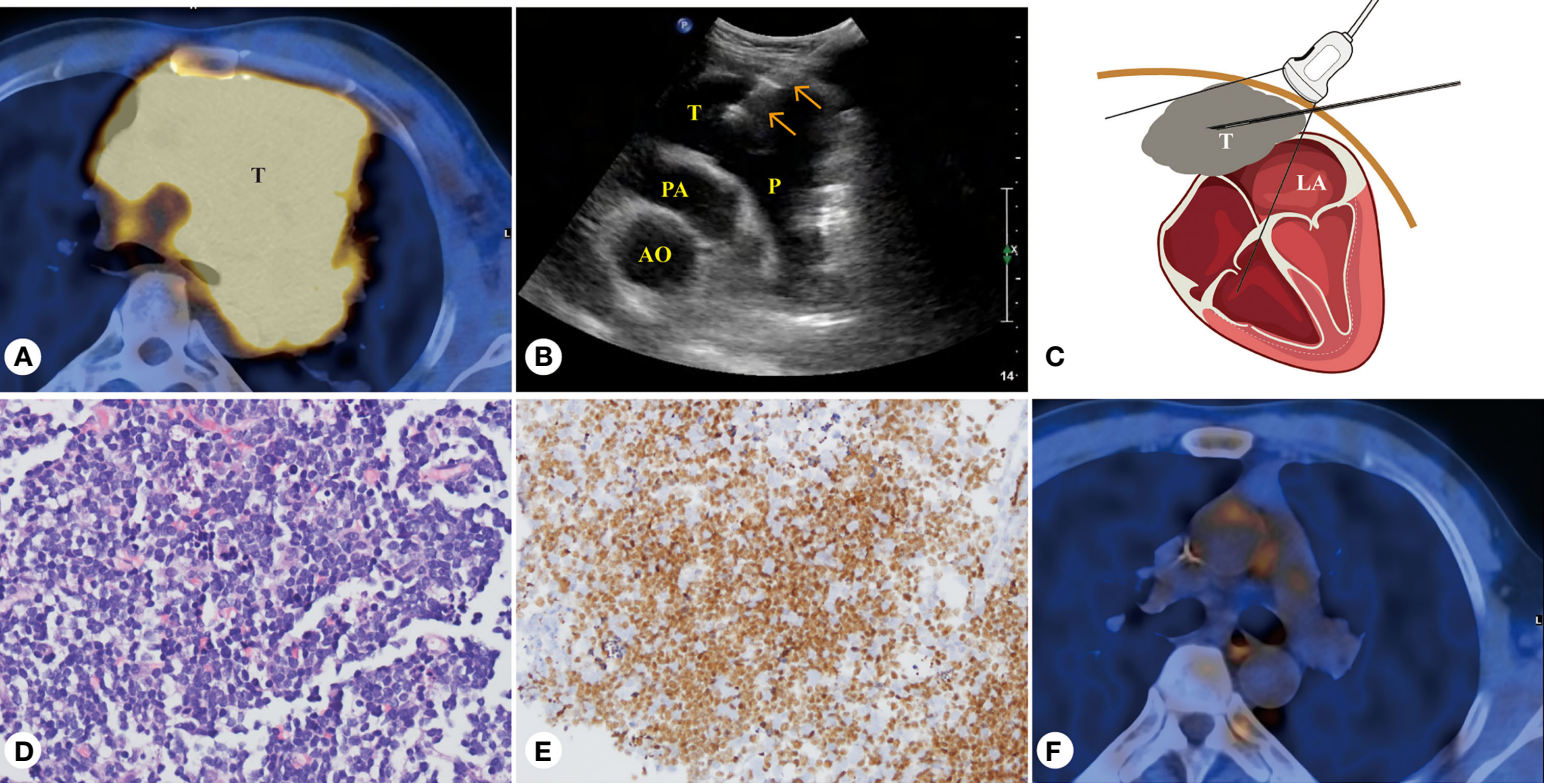
Forty-three-year-old male patient, T-cell blastoma of the heart. **(A)** PET/CT presentation of a mediastinal mass with a maximum SUV of 8.7 at the base of the heart and the pericardium. **(B)** PUS-CNB of the pericardial mass at the base of the heart from the left third intercostal space. **(C)** Schematic diagram of PUS-CNB. **(D)** The microscopic features showed diffuse patches of small- to medium-sized atypical lymphocytes (H&E, × 400). **(E)** Immunohistochemically, the tumor cells nuclei were strongly positive for TdT (× 200). **(F)** After four courses of chemotherapy, PET/CT showed that the T-cell blastoma lesion disappeared. AO, aorta; PA, pulmonary artery; P, pericardial space; T, tumor.

Pathology findings: Except for one failed case, pathology samples were successfully obtained in the remaining seven cases (87.5%), which showed three cases of diffuse large B-cell lymphoma (DLBCL) (shown in **Figure 1**), two cases of pericardial mesothelioma (shown in **Figure 2**), one case of T-cell-blast lymphoma (**Figure 3**), and one case of ectopic thymoma. The ectopic thymoma was surgically resected, while two cases of pericardial mesothelioma were treated with palliative care due to severity, and the remaining four cases of lymphoma were treated with chemotherapy, and the lesions disappeared in two patients after four cycles of chemotherapy. Specific clinical data are presented below in **Table 1**.

**Table 1:** Clinical data of PUS-CNB of cardiac tumors.

Case	Sex	Age	Location	Puncture Route	Final Pathology	Treatment Modality and Efficacy
1	Male	65	Posterior left atrial wall to the basal segment of the posterior left ventricular wall, left atrial cavity and interatrial septum	Left of the apex towards the base	DLBCL	Disappearance after chemotherapy
2	Female	67	Right ventricular lateral wall to pericardium	Apical right side	DLBCL	Shrinkage after chemotherapy
3	Female	83	Right ventricular lateral wall to intrapericardial	Apical right side	DLBCL	1 course of chemotherapy, intolerant
4	Male	43	Cardiac base great vessel and pericardium	Third rib space parasternal	T-cell blastocytic lymphoma	Disappearance after chemotherapy
5	Male	24	Visceral pericardium diffuse thickening	Apical part	Malignant mesothelioma	Palliative care
6	Female	60	Visceral pericardium diffuse thickening	Apical part	Malignant mesothelioma	Palliative care
7	Male	87	Apical pericardium	Apical part	Ectopic thymoma	Surgical incision
8	Female	74	Anterior right ventricular intrapericardial	Apical part	Biopsy failure	Preparation for surgical biopsy

### PUS-CNB of intracardiac combined with extracardiac tumor

The previous cases describe the experience of cardiac tumor biopsy, below we present cases where PUS-CNB is used on patients with cardiac combined with extracardiac tumor.

**Figure 4:**
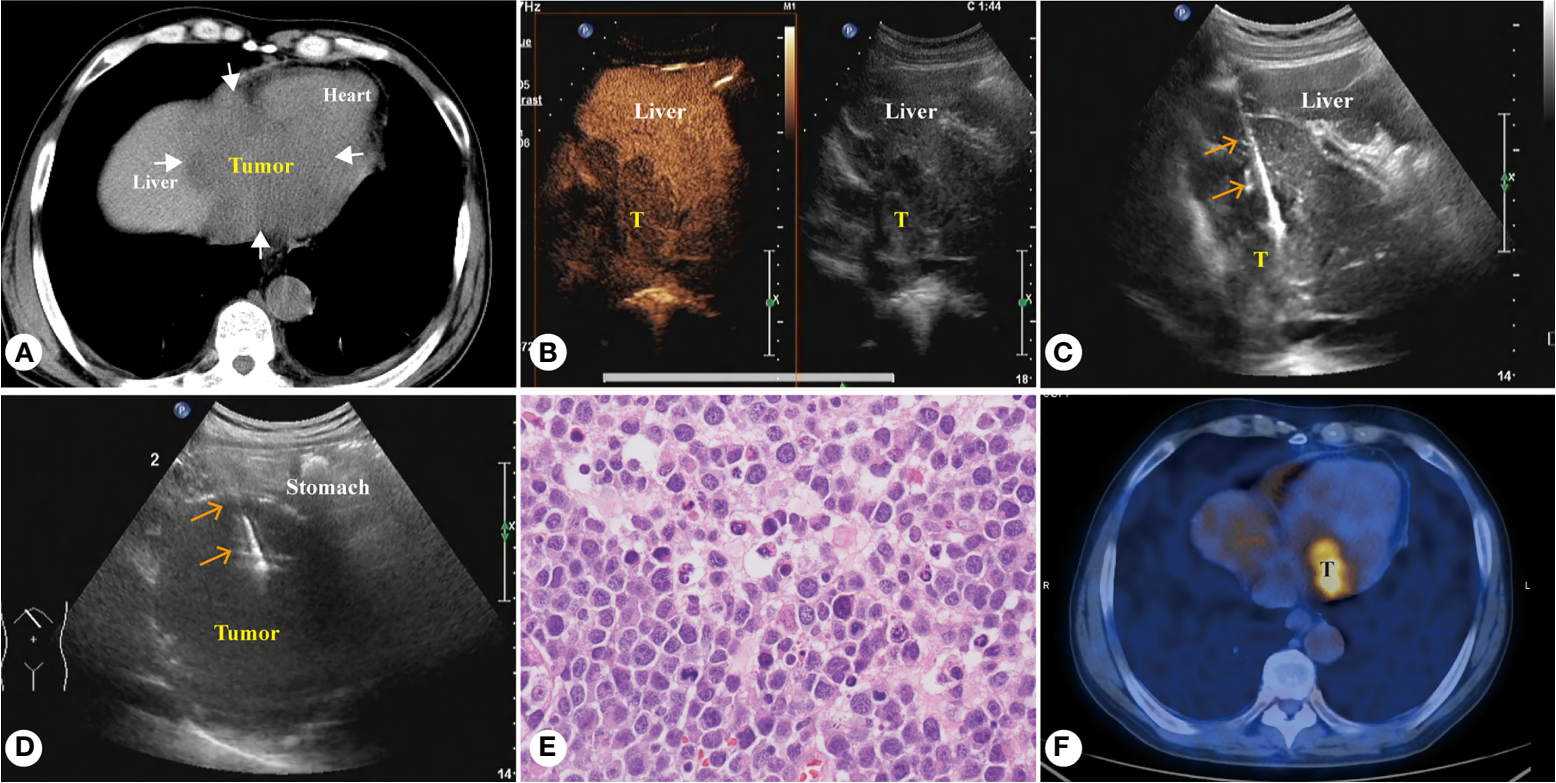
Sixty-five-year-old male patient, diffuse large B-cell lymphoma **(A)** CT showed a huge homogeneous hypodense mass involving the left liver, gastric fundus and heart. **(B)** Enhanced ultrasound showed intrahepatic homogeneous low enhancement tumor. **(C)** PUS-CNB of the left liver tumor. **(D)** PUS-CNB of the gastric fundus tumor. **(E)** The microscopic features showed diffuse patches of large atypical lymphocytes with many eosinophilic cytoplasm (H&E, × 400). **(F)** Tumor was significantly reduced after five courses of chemotherapy, but still active.

Four patients with intermural and pericardial tumors complicated with multiple systemic lesions were selected for PUS-CNB procedure at the most biopsy-prone sites, and the puncture times ranged from 8 to 17 (13.0 ± 2.9) min. The success rate of pathological sampling was 100% (4/4), and the pathological results showed that three cases were lymphoma and 1 case was thymoma. For the thymoma case, radiotherapy was administered after surgical resection of thymoma. For the lymphoma cases, one 65-year-old patient with systemic multiple lymphoma had experienced heart lesion disappearance after chemotherapy and died of gastric cancer 3 years later (**Figure 4**); one patient with lymphoma was in severe condition and chose palliative treatment. Specific clinical information is shown in **Table 2**.

**Table 2:** Patient data for PUS-CNB of extracardiac lesions.

Case	Sex	Age	Biopsy Location	Cardiac Tumor Location	Final Pathology	Treatment Modality and Efficacy
1	Male	65	Tumor of left liver and gastric fundus	Pericardium and pericardium	DLBCL	Lesion shrinkage after chemotherapy
2	Female	66	Swelling on the back of the left hand	Myocardium at the right atrioventricular junction	DLBCL	1 course of chemotherapy, intolerant
3	Male	31	Enlarged lymph nodes in the neck	Middle and lower ventricular septum and left ventricular anterior and lateral walls	DLBCL	Palliative care
4	Male	73	Mediastinal masses	Lateral wall of the right atrium to the pericardium	Thymoma	Post-surgical radiotherapy

### Exploratory thoracotomy biopsy

**Figure 5:**
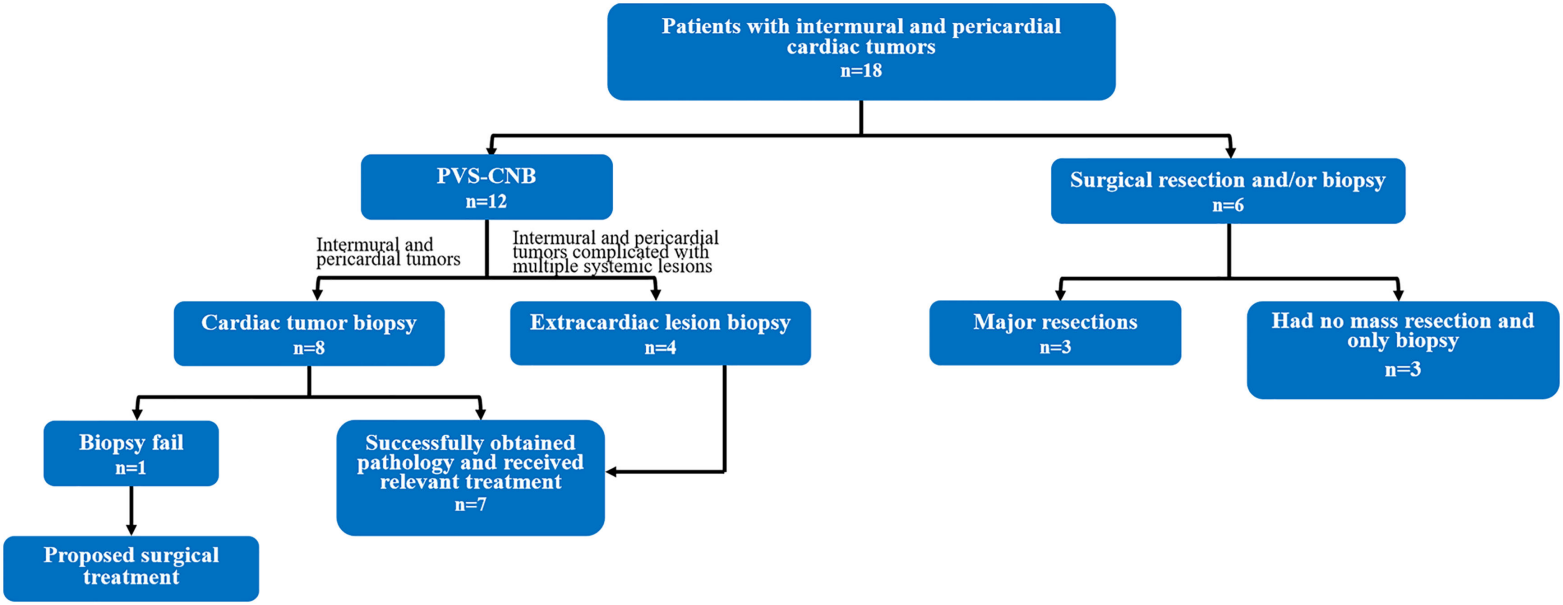
A schematic that demonstrate the procedure and/or the instrument setup.

Six cases were selected for direct surgical resection or biopsy, and the length of surgical incision was 16–20 cm. Three cases had no mass resection and only biopsy; three cases had major resections. The hemorrhage volume ranged from 100 to 500 (210 ± 148.8) ml, and the operation time ranged from 77 to 110 (89.5 ± 10.9) min. The condition of cases 2 and 3 aggravated after open-heart surgery and the treatment was abandoned. The specific clinical data are shown in **Table 3**. Differences between PUS-CNB and surgical resection are shown in **Table 4**. We drew a schematic (**Figure 5**) that demonstrates the procedure.

**Table 3:** Clinical information of patients treated for cardiac tumor surgery.

Case	Sex	Age	Location	Surgical Indications	Procedure Efficacy	Time	Final Pathology
1	Female	42	Visceral pericardium diffuse thickening	Constrictive pericarditis preparation for stripping	Intraoperative transfer biopsy	77 min	Malignant mesothelioma
2	Female	37	Left atrium and base of the heart	Left atrial mucinous tumor	Intraoperative transfer biopsy	85 min	Angiosarcoma
3	Female	40	Right atrium, base of the heart and pericardium	Cardiac tumor obstruction	Major incision	95 min	Granulocytic sarcoma
4	Female	46	Pericardium at the base of the heart	Mediastinal tumor involving the pericardium	Post-operative chemotherapy	110 min	Thymoma
5	Male	81	Right ventricle and anterior right ventricular wall	Right ventricular outflow tract stenosis	Post-operative chemotherapy	90 min	DLBCL
6	Female	69	Right atrial lateral wall to pericardium	Biopsy of pericardial masses	Chemotherapy after biopsy and operation	80 min	DLBCL

**Table 4:** Differences between PUS-CNB and surgical resection.

	Dominant position of biopsy	Time consumed (min)	Bleeding volume (ml)	Recovery time of wound	Length of stay in hospital (days)	Effective rate	Limitations
PUS-CNB	Cardiac wall type and pericardial tumor	15.6 ± 3.0	No obvious hemorrhage	The pain disappeared 1 h after the surgery	3	0.875	Potential for bleeding due to limited position of biopsy
Surgical biopsy	All positions	89.5 ± 10.9	210 ± 148.8	Wound healing time (8.8 ± 0.7 days)	12	0.667	Larger wounds lead to slower healing

## Discussion

For cardiac tumors, it is vital to gain an initial perspective through transthoracic Doppler echocardiography (TTE). TTE is the preferred screening modality for cardiac tumors, allowing initial determination of the malignancy of the tumor by its location, size, shape, activity, and affected regions. Seventy-five percent of cardiac myxoma have been reported to occur in the left atrium (11). In general, most cardiac tumors involving the right atrial wall should be considered malignant, and the right atrial wall is a rare site for benign tumors (12). Moreover, primary tumors located in the pericardium are generally malignant (13), and all five cases of tumors involving the right atrium and five cases of primary tumors in the pericardium in our group were malignant, which is consistent with previous studies. In addition, when a cardiac mass is combined with the presence of pericardial effusion, it should be considered as malignant tumor (14, 15). Integrating previous studies and the present study, it is revealed that intermural and pericardial primary cardiac tumors are basically malignancies, and if the tumor is combined with pericardial effusion, it further increases the diagnostic confidence.

CT and MRI are widely accepted methods for further diagnosis of cardiac tumors. MRI is the imaging method of choice for assessing tumor extension and tissue characteristics as it combines high spatial resolution with superior soft tissue contrast and possesses the unique advantages in identifying the nature of cardiac tumors. Malignant tumors appear on MRI as large, non-tipped masses with irregular, lobulated morphology that may invade the myocardium, pericardium, and adjacent extracardiac structures and are poorly demarcated from surrounding tissues (14). Enhanced MRI can identify the nature of cardiac tumors using first perfusion differences (16). PET/CT can differentially diagnose between benign and malignant cardiac tumors using the difference in cardiac or pericardial uptake of 2-[18F]-fluoro-2-deoxy-D-glucose (2-[18F]-fluoro-2-deoxy-D-glucose, FDG) (17, 18). However, imaging is not a pathological diagnosis after all, and there are cases of misdiagnosis. In our two cases of cardiac tumors, One of the patients undergoing thoracotomy was malignant mesothelioma. Preoperative imaging showed mild diffuse thickening of pericardium wall, and no increase in cancer scale, so it was misdiagnosed as constrictive pericarditis; one case of hemangiosarcoma was misdiagnosed as a common atrial myxoma on preoperative imaging as a solid mass protruding into the left atrium. Correction has been made in the revised version. The preoperative misdiagnosis of imaging resulted in the inability to perform the surgery normally after open-heart surgery. If the pathological diagnosis of tumor can be obtained preoperatively, unnecessary surgery can be avoided or the surgery can be planned better. Therefore, in this condition, biopsy becomes a necessary assistance before operations.

Biopsy of cardiac tumors has undergone improvements through the development of surgical biopsy, direct percutaneous biopsy, and transcatheter biopsy. It is known that traditional surgical open-chest biopsy is quite traumatic and the procedure is only performed when noninvasive biopsy fails or when the tumor causes obstruction and heart failure (19). In our research group, there was only one case of open chest exploration and biopsy with a wound length of 16 cm, which took 80 min and required not only general anesthesia, but still required a long postoperative healing time.

The advent of transcatheter biopsy was a major change that occurred in the history of myocardial biopsy (20) and has advantages for biopsy of intracardiac masses; the advantages includes that multiple specimens can be obtained without general anesthesia or open chest operations. Nevertheless, the procedure is more time-consuming, and with fine needle aspiration puncture, insufficient amount of biopsy tissue is obtained and posed high requirements for the pathologist; in addition, the transcatheter biopsy carries the risk of some complications, such as embolism, arrhythmias, valve damage, and pneumothorax (21, 22). Another scholar reported the use of endobronchial ultrasound bronchoscopic fine-needle aspiration technique (EUS-B-FNA) for biopsy of cardiac tumors near the esophagus (23), but restricting biopsy area.

The use of PUS-CNB for cardiac tumor has only been reported by a few researchers. Heenan et al. (24) reported a case of percutaneous transatrial fine needle aspiration of right atrial tumor through a substernal approach guided by ultrasound, with good surgical tolerance and no immediate or late complications. Kamal Gupta et al. (25) reported a case of this technique for pericardial tumor aspiration biopsy. Liwen Liu et al. (26) reported experience with the application of ultrasound-guided percutaneous transthoracic radiofrequency ablation for hypertrophic cardiomyopathy. Some scholars also reported case experience of CT fluoroscopy and CT-guided percutaneous transthoracic biopsy of left posterior atrial wall tumors and right ventricular outflow tract tumors with high accuracy and no complications (27, 28).

This study is the first reported experience of 12 cases of percutaneous ultrasound-guided core needle biopsy of cardiac tumors or extracardiac tumors for the diagnosis of intermural and pericardial tumors in the heart, and the results showed that this method is effective in obtaining pathological diagnostic information (success rate 91.7%).

As the success rate rests at 91.7%, it is worth noting that the cases of failed sample retrieval may be related to the failure to puncture the active tissue; therefore, it is recommended that in the future, interventionalists need to combine contrast-enhanced ultrasonography and e-MRI findings to locate the active site in a precise manner.

PUS-CNB is a real-time image-guided biopsy procedure that reduces the risk of cardiac complications compared to surgical biopsy, with no significant complications such as bleeding, infection, or arrhythmias. When combined with a large amount of pericardial fluid, pericardium catheter drainage is performed immediately after biopsy to facilitate the observation of the presence or absence of hemorrhagic fluid flow.

According to literature, secondary cardiac tumors are more common and their incidence is 30 times higher than that of primary cardiac tumors (29), and among them, the most common metastatic tumors are melanoma, lung cancer, breast cancer, lymphoma, and leukemia (30), with the most common cases as lymphoma in our case. When cardiac tumors are detected by ultrasound, it is important to perform a full body examination of the patient with the help of PET/CT, MRI and other imaging methods to exclude metastases. If multiple foci located either to the interior or exterior of the heart are found, biopsy of more easily biopsied lesions outside the heart is recommended. After obtaining pathology tissues, if diagnostic chemotherapy is taken, when the intra- and extra-cardiac tumors are found to respond consistently to drugs, then it is presumed to be homologous; conversely, if the intra-cardiac tumors do not respond to chemotherapy drugs, then they are considered to be independent tumors and other biopsy or surgical methods are planned. In our two patients with diffuse large B-cell lymphoma with multiple foci throughout the body, the intracardiac masses disappeared or shrank with chemotherapy after obtaining pathology from extracardiac lesions by puncture biopsy, which indirectly confirmed that the intracardiac tumors were metastatic lymphomas.

Different biopsy modalities have their advantages and disadvantages, and their clinical selection depends on the instrumentation, institutional experience, and individual differences in the patient’s condition. Comparing and analyzing previously mentioned biopsy modalities, we conclude that the advantages of PUS-CNB are as follows: ① Protected safety: when choosing the apical area of heart as the puncture path, the oblique cutting method is used (fifth and sixth rib space is chosen according to the location of the mass), so that the puncture needle is parallel to the myocardial wall as much as possible; when choosing the left parasternal side as the puncture path (third and fourth rib space), cases are strictly screened and masses with a safe distance are chosen, which can avoid the case where the biopsy needle penetrates the myocardium or large blood vessels leading to hemorrhage; the operation process can be monitored in real time to ensure that the operation process is safe and controllable, and can be multi-planar imaging, so that the operator can work with confidence. ② Relatively simple operation process: Experienced interventionalists can screen a suitable case by conventional ultrasound and perform the PUS-CNB without too much pre-operative preparation; the operation time for puncture biopsy is short, with a minimum of 9 min and an average of 15.6 ± 3.0 min. ③ Precise accuracy: the application of 18G coarse needle biopsy can obtain a sufficient amount of pathology tissue, which can improve the diagnostic accuracy compared with the fine needle aspiration biopsy method. ④ Radiation-free, less traumatic, and low cost: compared with fluoroscopy or CT-guided aspiration biopsy, the radiation-free imaging of ultrasound manifests its advantage; puncture sampling is basically non-invasive to the patient. Because of the simplicity of the procedure, the economic consumption incurred is also quite low.

PUS-CNB of cardiac tumors also has its limitations: (1) High requirements for interventionalists: the beating of the heart and cardiac tumor increase the difficulty of lesion display and puncture and inexperienced interventionalists are incompetent. (2) Limited puncture field of view: it is difficult to biopsy the lesions far away from the chest wall and those interfered by bone and lung air, so the number of suitable clinical cases is limited.

Objectively, PUS-CNB of cardiac tumors is not without risks, and its potential complications include pericardial tamponade, pneumothorax, induced arrhythmias, and vascular injury, especially of the internal thoracic and epicardial arteries (31). Yet, the complications were not present in our patient cases. To ensure proper safety and caution, postoperative observation for 1 h and close cooperation with the clinical department are required to address any complications as soon as they are identified.

There are some limitations to our study. Cardiac tumors are uncommon in clinical practice, and malignant tumors of the heart are relatively rare; therefore, the study results are limited by the small sample size, inherent biases and variations were inevitable, and a larger sample size study should and would be conducted in the future for further verification.

## Conclusion

Our study yielded interesting early results: on the basis that echocardiography is the preferred diagnostic tool for cardiac tumors, percutaneous ultrasound-guided core needle biopsy has been clinically validated as a safe, effective, and feasible technique to effectively diagnose specific cardiac intermural and pericardial tumors, optimize clinical treatment strategies, and avoid unnecessary open-heart surgery. Due to the small sample size of this study, a multi-center study with an expanded sample is needed to further investigate the factors affecting percutaneous ultrasound-guided core needle biopsy for cardiac tumors.

## Data availability statement

The raw data supporting the conclusions of this article will be made available by the authors, without undue reservation.

## Ethics statement

The studies involving human participants were reviewed and approved by the Institutional Review Board approval of Fujian Provincial Hospital. Written informed consent was obtained from all patients in this study.

## Author contributions

S-sW, Y-cZ, Z-lH, and YX planned the study, recruited and followed the patients, and wrote the paper. SC contributed in writing the paper. Y-cL contributed in picture editor. All authors contributed to the article and approved the submitted version.

## Funding

This study has received funding by Fujian Natural Science Fund (Grant No. 2020J011090).

## Conflict of interest

The authors declare that the research was conducted in the absence of any commercial or financial relationships that could be construed as a potential conflict of interest.

## Publisher’s note

All claims expressed in this article are solely those of the authors and do not necessarily represent those of their affiliated organizations, or those of the publisher, the editors and the reviewers. Any product that may be evaluated in this article, or claim that may be made by its manufacturer, is not guaranteed or endorsed by the publisher.
